# The Clinical Utility of Vestibular-Evoked Myogenic Potentials in the Diagnosis of Ménière’s Disease

**DOI:** 10.3389/fneur.2017.00415

**Published:** 2017-08-15

**Authors:** Maxime Maheu, Jenny Marylin Alvarado-Umanzor, Audrey Delcenserie, François Champoux

**Affiliations:** ^1^School of Speech Language Pathology and Audiology, Université de Montréal, Montreal, QC, Canada; ^2^Psychology, Université de Montréal, Montreal, QC, Canada

**Keywords:** Ménière disease, endolymphatic hydrops, occular vestibular-evoked myogenic potential, cervical vestibular-evoked myogenic potentials, vestibular-evoked myogenic potential

## Abstract

Ménière’s disease (MD) is a condition that has been proposed over 150 years ago, which involves audiological and vestibular manifestations, such as aural fullness, tinnitus, vertigo, and fluctuating hearing thresholds. Over the past few years, many researchers have assessed different techniques to help diagnose this pathology. Vestibular-evoked myogenic potential (VEMP) is an electrophysiological method assessing the saccule (cVEMP) and the utricule (oVEMP). Its clinical utility in the diagnosis of multiple pathologies, such as superior canal dehiscence, has made this tool a common method used in otologic clinics. The main objective of the present review is to determine the current state of knowledge of the VEMP in the identification of MD, such as the type of stimuli, the frequency tuning, and the interaural asymmetry ratio of the cVEMP and the oVEMP. Results show that the type of stimulation, the frequency sensitivity shift and the interaural asymmetry ratio (IAR) could be useful tool to diagnose and describe the evolution of MD. It is, however, important to emphasize that further studies are needed to confirm the utility of VEMP in the identification of MD in its early stage, using either bone-conduction vibration or air-conduction stimulation, which is of clinical importance when it comes to early intervention.

## Introduction

Ménière’s disease (MD) is a condition that has been proposed over 150 years ago, which involves episodic audiological and vestibular manifestations (1), such as spontaneous vertigo, tinnitus, aural fullness, and fluctuation in hearing thresholds (2). Based on the presence or absence of these symptoms, the Committee on Hearing and Equilibrium (3) defined four different diagnostic categories as follows: certain, definite, probable, and possible. However, an amendment to the diagnostic categories was recently made so that only two diagnostic categories are now accepted: definite and probable (2).

Although several studies have examined this pathology, little is known about the exact cause(s) of MD. The most supported pathophysiological mechanism of this disease is endolymphatic hydrops (EH). EH is a disorder where excessive endolymph accumulates in the cochlea and vestibular organs (2, 4) and has been found to be a strong marker of MD (5). Histopathological studies indeed showed that EH is present in 98.8–100% of all confirmed cases of MD (5, 6). Interestingly, studies have shown that the cochlea and the saccule are the structures that are the most commonly affected by EH, followed by the utricule and semi-circular canals (7–10). Indeed, EH does not seem to affect inner ear structures equally, so that pars inferior structures (cochlea and saccule) have been shown to be more susceptible to present EH (7). Based on this proposed pathophysiological mechanism, few studies have investigated the influence of diuretics (glycerol and furosemide) on hearing and vestibular tests. However, most of these studies have investigated the effect of diuretics on the functioning of the cochlea (8, 9, 11) and the semi-circular canals (8, 12, 13).

In terms of MD’s diagnosis, research generally shows that there is no objective gold standard measure. Although it has been shown that electrocochleography and magnetic resonance imaging could both be useful in order to detect EH (14–16), these measures have mainly focused on the cochlea. Other studies have used common vestibular measures such as caloric and video head-impulse test as well as vestibular-evoked myogenic potentials (VEMP) to differentiate MD from healthy ears (17, 18).

The VEMP is a clinical evaluation method that assesses the integrity of the saccule (cVEMP) and the utricule (oVEMP) (19). The cVEMP is an inhibitory response measured from the ipsilateral sternocleidomastoïd muscle with a positive peak occurring around 13 ms (P1) and a negative peak around 23 ms (N1) (19, 20). The oVEMP is an excitatory response measured from the contralateral inferior oblique muscle with a negative peak occurring around 11–12 ms (N1) and positive peak occurring around 18 ms (P1) (21). As mentioned earlier, since EH affect more specifically the cochlea and the otolith organs, the VEMP could be a promising tool in the identification of EH and, thus, in the diagnosis of MD.

The main objective of the present review is to determine the current state of knowledge of the VEMP in the identification of EH. We will review the use of glycerol and furosemide on the VEMP responses and parameters, such as the type of stimuli, the frequency tuning, and the interaural asymmetry ratio in the identification of EH. Since these parameters were the most often used in the literature, they will be the focus of the present review.

## Furosemide and Glycerol

Endolymphatic hydrops is a phenomenon that has been described as excessive endolymph that accumulates in the inner ear (2, 4). Therefore, it has been suggested that diuretics, such as furosemide or glycerol, could alleviate EH pressure (22).

In line with this assumption, a small number of studies have focused on possible VEMP modifications induced by glycerol (23–25) and furosemide (26, 27). These studies have shown a significant improvement in cVEMP amplitude ranging from 39 to 44% of MD participants following administration of the diuretic but no significant modification of P1 and N1 latencies (24, 25). Interestingly, Seo et al. (27) suggested that this test might be useful to detect EH in the contralateral ear of MD participants. In fact, Ban et al. (24) demonstrated modifications of cVEMP amplitude in 17.9% of MD participants’ unaffected ears. The latter study is the only one to have controlled for EMG level. This is of importance because EMG level can induce significant amplitude modifications that could alter the interpretation of the diuretic’s effect on cVEMP response.

Finally, the combination of VEMP and diuretic administration (glycerol or furosemide) seems to be a promising field to explore. To date, little is known about the effect of diuretics on the cVEMP response and no study has evaluated their on oVEMP responses. Further studies should, therefore, investigate possible modifications of oVEMP responses and differences in VEMP responses using air- or bone-conduction stimulation.

## Type of Stimuli

In humans, VEMP responses can be elicited through various stimuli. Air-conduction (ACS), bone-conduction vibration (BCV), or galvanic stimulation have been shown to trigger oVEMP and cVEMP responses in normal healthy participants and several clinical populations [see Ref. (19, 20) for a complete description]. Since ACS and BCV are most frequently used in clinical settings, it is important to address the differences that exist between these two stimuli in MD population.

Previous studies have shown that MD differently affects ACS and BCV. First, cVEMP and oVEMP amplitudes have been shown to be significantly larger using BCV than ACS (28). Second, cVEMP and oVEMP response rate was found to be significantly lower using ACS rather than BCV in the affected ear (28–30). These authors observed that, using ACS, 45–80% of individuals with MD demonstrated a reduced or absent response to cVEMP. However, only 10–30% of individuals with MD showed such abnormal response using BCV, which could indicate that BCV could assess the residual otolith function in MD participants.

Of interest, two studies investigated the asymptomatic ear of individuals with unilateral MD. Wang et al. (30) showed that ACS or BCV could both be used to elicit a 100% response rate for cVEMP and oVEMP. However, Huang et al. (29) showed that differences exist between ACS and BCV when it comes to the asymptomatic ear. They found an abnormal response rate of 15 and 40% for cVEMP and oVEMP, respectively, when using ACS. With BVC, this abnormal response rate was found to be equivalent to 0%, this for both cVEMP and oVEMP. The poor description of the MD participants in Wang et al.’s (30) study prevents us from determining if sample differences could explain the discrepancy in the results. However, these last results suggest that BCV could be more effective in evoking VEMP responses in individuals with MD.

One hypothesis to explain a higher BCV response rate might be related to the interaction between the pathophysiology of MD on the macule and the fact that ACS and BCV have different transduction mechanisms (31). First, MD seems to selectively affect type II hair cells (32, 33), whereas VEMP response has been shown to depend heavily on type I hair cells (34). The fact that ACS only activates a subset of the neurons normally activated by BCV (19) could contribute to explain the larger VEMP responses found using BCV stimulation mode in comparison to ACS. Since the most common stimulation mode for VEMP in clinical setting is ACS, and knowing that this often leads to absence of VEMP responses, experimenters should be careful and use BCV to better support their findings/diagnosis. Further studies are, however, needed to examine the impact of stimulation mode during the different stages of MD, which could be useful to determine the extent of vestibular lesions.

## Frequency Tuning Curve

In healthy individuals, VEMP responses have been shown to be more sensitive to low frequencies. Indeed, for both oVEMP and cVEMP, a 500 Hz tone-burst evoke larger amplitudes, lower thresholds, and a higher response rates than other frequencies (35, 36). However, the literature suggests that MD might influence the frequency sensitivity of the VEMP response. First, the studies that evaluated the frequency tuning of cVEMP responses all suggest the presence of a shift from lower frequencies (500 Hz) to higher frequencies (750 Hz and 1 kHz), which could be useful in identification of MD [e.g., (37–44)]. More specifically, Sandhu et al. (40) observed a shift in frequency sensitivity from 500 Hz (healthy individuals) to 750 Hz (individuals with MD). Interestingly, these authors pushed the analyses further by examining potential differences between individuals with definite and probable MD, as determined by AAO-HNS 1995, the Committee on Hearing and Equilibrium guidelines (3). They found that the shift in frequency sensitivity is found only for individuals with definite MD, but not in individuals with probable MD or controls. No significant differences in frequency sensitivity were found between individuals with probable MD and controls, neither between the unaffected ear of MD participants and controls. In terms of oVEMP, studies have found similar results. However, the shift in frequency sensitivity seems to be increased to higher frequencies than the cVEMP. In fact, the oVEMP response seems to be maximized at 1,000 Hz in groups with MD (43, 45). Sandhu et al. (40) supported that individuals with definite MD, differed significantly from healthy participants when comparing oVEMP responses to frequencies between 750 and 2,000 Hz.

Although the amplitude of VEMP response to multiple frequencies have often been examined in the literature, several studies also examined VEMP frequency sensitivity using 500/1,000 Hz amplitude ratio as a possible criterion to identify MD (39, 42–44). The interpretation of this ratio is as followed: (1) if the 500/1,000 Hz amplitude ratio is elevated, the saccule is more sensitive to 500 Hz as opposed to 1,000 Hz and (2) if the ratio is low, this would mean a higher sensitivity of the saccule to 1,000 Hz. The results of these studies agreed that the 500/1,000 Hz ratio was able to distinguish between affected and unaffected ears of individuals with MD (31), but also between MD and healthy individuals (39, 42–44). This is in line with the previous hypothesis that there is a shift in sensitivity to higher frequencies for VEMP results.

However, important age differences existed in some of these studies between individuals with MD and controls, where MD participant are significantly older. Since age is known to affect frequency tuning (46), these differences could instead be due to normal aging, instead of EH. Nevertheless, studies that controlled for age showed that it could influence frequency tuning in controls and in the unaffected ear of individuals with MD, but not in ears affected by MD (41–44).

One hypothesis that has been put forward to explain VEMP response’s frequency specificity shift is the increased impedance induced by EH (42). Jerin et al. (42) stated that EH may increase stiffness and, therefore, reduce low-frequency transmission in the inner ear, explaining the increase in frequency specificity. This phenomenon can explain the higher amplitudes observed at higher frequencies in individuals with MD and the reduced response at 500 Hz. One, however, has to be cautious with this hypothesis since normal healthy aging and semi-circular canal dehiscence are known to induce a similar frequency shift (46, 47). Age norms are, therefore, required when analyzing frequency tuning. Interestingly, to our knowledge, no studies looked at BCV VEMP frequency tuning in individuals with MD. Further studies should, thus, examine if any difference exist between ACS and BCV VEMP frequency tuning in this population.

## Interaural Asymmetry Ratio (IAR)

Another parameter of interest is IAR. In healthy individuals, VEMP amplitudes between ears are usually relatively symmetrical. Therefore, when IAR is abnormally elevated, it is possible to identify the ear with the lowest function. However, there is no consensus between studies that examined IAR in MD participants, as some observed an asymmetry explained by a reduced response (48, 49) or an enhanced (50, 51) response in the affected. These conflicting results can, however, be at least in part, explained by sampling differences. Indeed, most of Young et al.’s (50) participants were in the early stages of MD, whereas those of Kuo et al. (48) were at stages III and IV of the disease. In line with these studies, Young et al. (49) assessed participants with unilateral MD using ACS cVEMP. They found a significant positive correlation between IAR and MD’s stages. They suggested that enhanced cVEMP response in the affected ear is often associated with the beginning of MD and, as MD evolves, a decrease in amplitude in the affected ear is observed. Enhanced VEMP responses in early MD has also been found using BCV oVEMP (52). Wen et al. (52) found a significant negative correlation between enhanced oVEMP response and the increasing stages of MD. However, this trend was not observed using BCV cVEMP. Indeed, Manzari et al. (51) investigated 15 participants with definite unilateral MD who were at the early stages of the disease. The participants were assessed during both quiescent periods and MD attacks using BCV cVEMP and BCV oVEMP. The results showed a dissociation of the VEMP responses during attacks, where BCV oVEMP were enhanced and BCV cVEMP were reduced.

These results suggest that it might be possible to monitor MD stages using IAR. For example, a study examined patients who received a diagnosis of either sudden hearing loss or unilateral MD on a daily basis. They were assessed using ACS cVEMP and pure tone audiometry (50). The results showed that 7 participants with EH out of 10 had stable VEMP responses hearing thresholds along with significantly reduced cVEMP response to the affected ear. However, three participants with EH showed enhanced cVEMP response at the hydropic ear, associated with hearing thresholds fluctuations. Young (18) suggests that this enhanced cVEMP response could be attributed to the distension of the saccule’s walls, which comes in contact with the footplate. Kuo et al. (48), for example, found that two-thirds of their participants with unilateral MD (8 out of 12) had absent or reduced cVEMP responses in the affected ear 24 h after the MD attack. It is, however, important to note that, 48 h following the attack, four of the participants (33.3%) who had abnormal cVEMP responses had returned to normal. If we attributed the attack to a distension of the saccule due to EH, the results of augmented VEMP response could be explained by the distension of the saccule.

Overall, the literature shows that IAR could be useful to determine MD’s evolution. Indeed, this technique makes it possible to observe enhanced cVEMP responses in the affected ear during MD’s early stages and reduced cVEMP responses as the disease progresses. For example, Manzari et al. (51) observed a dissociation in VEMP responses between attack and quiescence periods of MD. Even though these results could be useful to better understand the pathophysiology of MD’s attack, more studies are needed before any conclusion can be made on the clinical usefulness of this parameter.

## Conclusion

Recent advances in VEMP responses analysis demonstrate the usefulness of this technique to identify the affected ear of individuals with MD. From the use of different stimulation mode (ACS or BCV), the use of various stimulation frequencies, and the analysis of interaural asymmetry ratios, VEMPs are an interesting tool to assess the MD’s progression. These findings could be related to mechanical changes induced by EH, a strong maker for this disease. It is, however, important to remain cautious when associating EH with MD, since EH could also be present in individuals who do not have an MD diagnosis (53). Therefore, VEMP findings in the diagnosis of MD should be analyzed in the light of the symptoms described by the patients, but also using the results of other evaluations. In terms of diagnostic efficiency, modifications in cVEMP amplitude following glycerol or furosemide administration, BCV stimulation, and frequency sensitivity shift appear to be better supported than IAR and, thus, should be considered first when MD is suspected. Finally, further studies assessing the impact of diuretics on VEMP response should focus on assessing oVEMP and examine whether specific differences arise from the use of BCV or ACS stimulation. Moreover, further studies are needed to evaluate the usefulness of VEMP, using either BCV or ACS, for the early identification and the development of a proper classification of MD, which is of clinical importance when it comes to early intervention.

## Author Contributions

MM and FC performed the review of the literature and wrote the paper. JA-U and AD revised the manuscript and provided comments. All authors discussed the results and implications and commented on the manuscript at all stages.

## Conflict of Interest Statement

The authors declare that the research was conducted in the absence of any commercial or financial relationships that could be construed as a potential conflict of interest.
